# Agronomic Evaluation of Sorghum Hybrids for Silage Production Cultivated in Semiarid Conditions

**DOI:** 10.3389/fpls.2017.01088

**Published:** 2017-06-22

**Authors:** Alexandre F. Perazzo, Gleidson G. P. Carvalho, Edson M. Santos, Higor F. C. Bezerra, Thiago C. Silva, Gildenia A. Pereira, Rosângela C. S. Ramos, José A. S. Rodrigues

**Affiliations:** ^1^Programa de Pós-graduação em Zootecnia, Universidade Federal da BahiaSalvador, Brazil; ^2^Programa de Pós-graduação em Zootecnia, Universidade Federal da ParaíbaAreia, Brazil; ^3^Departamento de Zootecnia, Universidade Federal da GoiásGoiânia, Brazil; ^4^Programa de Pós-graduação em Zootecnia, Universidade Estadual do Sudoeste da BahiaItapetinga, Brazil; ^5^Centro Nacional de Pesquisa de Milho e Sorgo, EmbrapaSete Lagoas, Brazil

**Keywords:** dry matter, forage, panicle, plant for silage, regrowth

## Abstract

The aim of this study was to study the agronomic traits of different *Sorghum bicolor* (L.) Moench hybrids for silage productionin semiarid conditions. It was a 1-year evaluation conducted in a randomized block design with 24 treatments and three replicates. The treatments were sorghum hybrids developed by the breeding program of “Embrapa Milho e Sorgo” (Brazilian Agricultural Research Corporation). The fresh matter yield (FMY) in the first cut varied from 22,643.56 to 44,033.15 kg/ha, with an average of 32,607.37 kg/ha, leading to the formation of two groups. Similar results were observed for the dry matter yield (DMY), in which the highest group yielded from 9,471.32 to 14,540.23 kg/ha dry matter (DM). For plant regrowth, there was an increase in the number of stems and a decrease in the amount of panicles. Two groups were formed for the accumulated dry matter yield (ADMY), averaging 14,217.91 kg/ha; the highest group showed mean values of 18,003.00 to 14,221.94 kg/ha. The evaluated sorghum hybrids exhibited satisfactory accumulated forage yields due to their high yield in regrowth, which indicates that they are suitable for use in animal production systems in semi-arid regions.

## Introduction

Sorghum is an important forage crop used in livestock systems in many regions around the world because of its adaptation to different environments (Sanchez et al., 2002; Fonseca et al., 2012; Amelework et al., 2015). In arid and semiarid regions, sorghum is an important source of roughage because of its morpho-physiological adaptations to the water stress that provide high dry matter yield (DMY) and adaptability in areas with uneven distribution of rainfall (Sankarapandian et al., 2013; Ahmeda et al., 2016; Rakshit et al., 2016; Su-jiang et al., 2016).

Beyond those characteristics, sorghum plants have is suitable for silage production (Brocke et al., 2014), because of its high concentration of soluble carbohydrates (Sankarapandian et al., 2013) low buffering capacity and high nutritional value (Lema et al., 2001; Kumar et al., 2015), all of which are essential for adequate lactic acid fermentation (Santos et al., 2013). It is important because silage making is one main method of forage conservation used to provide food for the animals during the drought period in tropical regions.

In this context, the evaluation of sorghum hybrids for silage production is a constant step in the breeding programs because different genotypes are developed over the years, which emphasize the need of their evaluation in different locations and years. According to Qu et al. (2014), plant breeders have focused on traits likely to affect its productivity, such as yield and/or forage quality. Thus, to define the average profile of the genetic material of each hybrid, it is necessary to characterize the hybrids through the morphological and productive characteristics, as the percentage participation and the chemical compositions of the major anatomical structures of the plant (Muturi et al., 2014).

One advantage of sorghum is its ability to regrow after the original culture in the field is cut, especially when fertilization is employed (Afzal et al., 2012). Therefore, it is possible to produce silage without the need for replanting sorghum. However, it is important to conduct an agronomic study at each cut due to the possible morphological and physiological changes in the plant that can affect the forage yield and other phenotypic traits, which might consequently modify the nutritional value and fermentation of the silage.

The objective of this study was to perform an agronomic evaluation of 24 sorghum hybrids at the first and second cuts.

## Materials and methods

The experiment was conducted at *Estaç*ã*o Experimental Pendência* (Experimental Station) of Empresa de Pesquisa Agropecuária da Paraíba S.A.—EMEPA (Agricultural Research Corporation of Paraíba State, Brazil), located in the city of Soledade, Paraiba State, Brazil (coordinates 7°8′18″ S and 36°27′2″ W; altitude 534 m).

Based on the Köppen classification, the climate of the region is a *Bsh* type (hot semi-arid), with rains from January to April, an average temperature of ~24°C, a relative humidity of approximately 68% and an average annual rainfall of 400 mm.

The experimental treatments consisted of 21 sorghum experimental hybrids and three commercial hybrids developed by the breeding program of *Embrapa Milho e Sorgo* (Brazilian Agricultural Research Corporation). We evaluated the first and second cuts of the following hybrids: 944007, 944056, 944040, 944009, 945015, 945019, 945020, 945023, 945026, 945021, 945027, 945022, 944043, 944033, 944034, 946007, 946015, 946016, 946013, 946042, 946043, BRS655, Volumax and BRS610. The hybrids were evaluated in a randomized-block design with 3 replicates.

Seeds were manually sown in 4.2 m^2^ (4.2 × 1.0 m) plots with 70 cm between rows. Thinning was performed 30 days after planting to maintain a density of 12 plants per linear meter. The soil was fertilized 15 days after sowing based on the chemical properties of the soil in the experimental area, using 50 kg nitrogen in the form of ammonium sulfate (Table 1). The crop was grown only under rainfed conditions.

**Table 1:** The chemical properties of the soil in the experimental area.

**pH**	**P**	**K^+^**	**Na**	**H^+^+Al^+3^**	**Al^+3^**	**Ca^+2^**	**Mg^+2^**	**SB**	**CEC**	**V**	**O.M**.
H_2_O	mg/dm^3^		cmol_c_/dm^3^							%	g/kg
6.27	70.96	215.69	0.2	3.30	0,00	7.05	2.25	10.0	13.35	75.28	10.94

*pH, potential of hydrogen; P, phosphorus; K^+^, potassium; Na^+^, sodium; H^+^+Al^+3^, potential acidity; Al^+3^, aluminum; Ca ^+2^, calcium; Mg^+2^, magnesium; V%, base saturation; CEC, cation exchange capacity; OM, organic matter; SB, Sum of bases*.

The plants were harvested when the grains reached the milky/dough stage. Two separate harvests were performed for the first cut, because the experimental hybrids reached harvest stage at different days. The length of the cycle of the first cut, from planting to harvest, was 71 and 80 days for the first and second harvests, respectively. At the first harvest, the following hybrids were collected: 944007, 944056, 944040, 944009, 945015, 945019, 945020, 945023, 945026, 945021, 945027, 945022, 944043, 944033, 944034, 946007, 946015, 946016, 946013, 946042, 946043, and BRS655; at the second harvest, only hybrids Volumax and BRS610 were included. Only one harvest was performed at the second cut, and the cycle length for the hybrids from the first cut to regrowth were 40 and 31 days for the first and second harvests, respectively. The evaluation cut was manually performed 10 cm above the soil surface with knives. For evaluation purposes, we considered the yields of two meters of furrow per plot by counting the number of plants per linear meter. The total accumulated rainfall during the two cycles was 635 mm.

At each cut, we evaluated the plant height (PH, m), stem diameter (SD, cm), number of tillers (NT), number of leaves (NL), average leaf size (ALS, cm), panicle size (PS, cm), fresh matter yield (FMY, kg/ha) and DMY (kg/ha), and the proportion of the components panicle, leaf blade and stem in the DM of the plant. The NT was determined as the number of tillers per cultivated linear meter and the total meters per hectare. The material collected from each plot was separated into panicles, leaf blades and stems, and each fraction was separately weighed. To estimate the DM content, a subsample of each fraction was dried at 65°C until a constant weight was reached. From these data, we assessed the proportion of plant components in g/kg for DM. Fresh matter yield, expressed as kg/ha, was calculated as the product between the yield of a cultivated linear meter and the total cultivated linear meters per hectare. Dry matter yield, expressed as kg/ha, was estimated as the product between the FMY and the DM content. The total fresh matter yield (TFMY) was estimated as the sum of the FMY obtained from the 2 cuts, and the total dry matter yield (TDMY) was estimated as the sum of the DMY obtained from the 2 cuts.

The results were subjected to variance analysis considering the effects of hybrids and blocks, and the Scott-Knott test (α = 0.05)was used to compare the means of each hybrid within each harvest, by using the SAEG software version 8.0 (Euclydes, 2004).

## Results

The NT was similar among the hybrids at both first and second cuts (*P* > 0.05). Difference (*P* < 0.05) was found for PH among the hybrids At the first cut, the group with the greatest heights ranged from 1.89 to 2.20 m, whereas the group with the lowest heights ranged from 1.86 to 1.54 m. In the second cut, the average PH decreased to 1.75 m. The second cut also formed 2 groups, with the taller group varying from 1.98 to 1.73 m and the shorter group ranging from 1.64 to 1.35 m. Hybrids showed different values of SD (*P* < 0.05) only at the first cut, with the highest values observed for the hybrids 3, 4, 7, 9, 10, 13, 18, 23 (>2.10 cm).

The number of leaves (NL) in the first cut varied (*P* < 0.05) from 9.83 to 5.33, with an average of 7.28. Four distinct groups were formed, and hybrids 23 and 24 had the highest values for this variable, at 9.83 and 9.33, respectively. With regards to the second cut, two groups were formed (*P* < 0.05), with the number of leaves ranging from 8.67 to 6.00 and an average 7.40 (**Table 5**). The variable average leaf size (ALS), in the first cut, was 57.94, ranging (*P* > 0.05) from 45.17 to 68.33 cm. In the second cut, the average varied (*P* > 0.05) from 41.00 to 56.50 cm, averaging 45.74 cm. A decrease in the ALS was observed for the second cut compared with the first cut. A significant difference (*P* < 0.05) was observed for panicle size (PS) among the hybrids, with the formation of two groups: a higher group, with values ranging from 33.17 to 27.67 cm, and a lower group, with values ranging from 27.00 to 22.33 cm. The hybrids did not differ (*P* > 0.05) in the second cut, but this variable decreased in the second cut (24.06 cm) compared with the first cut (26.56), which most likely influenced the decrease in the participation of panicles among the components of the sorghum morphology.

The percentage of leaf blades differed (*P* < 0.05) among the hybrids (**Table 4**) in the first cut. Hybrid 10 was notable, with 322.8 g/kg of leaf blades on its tiller, while the average of all genotypes was 138.0 g/kg. The stem percentages in the first cut was divided into two groups (*P* < 0.05): one with the highest values (534.1 to 694.5 g/kg, and other with the lowest values (524.1 to 322.8 g/kg). The percentage of panicles in the first cut differed (*P* < 0.05) among the hybrids, with an average of 325.8 g/kg, to form two groups. The higher group had values ranging from 430.9 to 337.2 g/kg, whereas the lower group had values ranging from 317.5 to 190.0 g/kg of the panicles in the plant.

Hybrids effect was observed (*P* < 0.05) on the percentage of leaf blades among the hybrids in the second cut, and two groups were formed. Hybrids 8 and 23 showed maximum values compared with the other hybrids, with 195.1 and 225.2 g/kg, respectively. There was no difference among the hybrids (*P* > 0.05) for the stem component in the second cut, and an increase in the amounts of stems for most hybrids was observed in the second cut, with a nearly 7% increase in the average (605.6 g/kg) compared with the first cut. The percentage values of panicles for the second cut did not differ (*P* > 0.05) among the hybrids; however, there was a 7.67% decrease in the average of this component for the second cut compared with the first, showing that the participation of panicles in the plant was affected by regrowth.

Dry matter content (*P* < 0.05) among the hybrids only for the first cut, with values ranging from 251.0 to 400.1 g/kg (Table 2). Four groups were formed, and the group with the highest DM content (above 370 g/kg) was composed of hybrids 2, 4, and 7. In the second cut, the hybrids showed similar DM content (*P* < 0.05) with an average of 260.7 g/kg. Fresh matter yield values differed (*P* < 0.05) among the hybrids only in the first cut. Under the conditions of the first cut, the FMY values ranged from 22,643.56 to 44,033.15 kg/ha (average 32,607.37 kg/ha), and two groups were formed: a more productive group, with FMY values above 30,000.00 kg/ha, and a less productive group, which averaged from 22,643.56 to 27,803.34 kg/ha. For the second cut, the average FMY was 14,607.38 kg/ha, with yields varying between 10,439.44 and 17,689.98 kg/ha. According to the average of each cut, the average FMY from the second cut was 43% of the average FMY from the first cut. Although a few hybrids were more productive in the first cut, they did not maintain their superiority during the regrowth period, as exemplified by hybrids 24 and 16, which reached FMY values above 40,000 kg/ha in the first cut but produced yields below the average for all hybrids in the second cut.

**Table 2:** Mean values of dry matter (DM) content, fresh matter yield (FMY) and dry matter yield (DMY) of 24 sorghum hybrids cultured in the semiarid region.

**Hybrids**	**DM (g/kg)**		**FMY (kg/ha)**		**DMY (kg/ha)**	
	**Cut 1**	**Cut 2**	**Cut 1**	**Cut 2**	**Cut 1**	**Cut 2**
944007	314.4c	225.0	22,643.56b	11,384.12	8,182.04b	2,600.53
944056	378.0a	317.8	27,803.34b	15,651.97	10,489.90a	4,900.15
944040	251.0d	258.3	33,790.32a	14,664.13	8,288.41b	3,827.57
944009	400.1a	262.5	35,861.90a	13,657.11	14,380.17a	3,594.71
945015	323.2b	267.0	34,080.44a	17,689.98	11,006.69a	4,674.22
945019	328.9b	248.7	23,355.67b	12,189.74	10,452.44b	3,035.75
945020	395.4a	268.0	34,442.48a	13,335.82	13,739.19a	3,570.23
945023	359.0b	267.2	30,414.40a	14,074.30	11,013.20a	3,794.80
945026	294.3c	269.8	25,563.92b	10,535.35	6,592.17b	2,860.14
945021	340.5b	243.6	26,264.04b	10,439.44	9,077.50b	2,549.35
945027	272.8d	252.1	30,354.46a	14,280.50	8,433.98b	3,570.56
945022	236.1d	258.7	32,272.59a	15,076.53	7,624.92b	3,866.74
944043	255.2d	258.7	25,880.41b	14,011.96	8,605.37b	3,647.65
944033	305.5c	273.0	39,597.46a	17,512.56	9,835.30a	4,790.76
944034	349.5b	286.2	34,508.42a	16,308.93	9,471.32a	4,653.87
946007	346.8b	259.3	41,254.25a	12,194.53	14,540.23a	3,170.26
946015	332.5b	257.0	33,970.14a	16,606.24	11,315.50a	4,265.10
946016	342.3b	268.4	31,876.98a	13,805.76	10,913.36a	3,704.98
946013	291.1c	305.5	32,620.25a	19,871.86	9,552.26b	5,942.66
946042	348.4b	265.3	36,588.39a	17,622.85	12,655.25a	4,660.75
946043	300.3c	260.1	38,362.66a	12,861.08	11,627.44a	3,342.74
BRS 655	325.2b	233.5	29,903.70a	16,822.03	9,738.70b	3,963.81
Volumax	313.1c	229.9	36,468.51a	14,807.99	11,441.15a	3,379.13
BRS 610	269.4d	220.6	44,033.15a	15,172.43	11,948.51a	3,339.84
Mean	319.7	260.7	32,607.37	14,607.38	10,455.21	3,821.10
CV^*^ (%)	11.25	10.18	25.10	27.62	29.08	27.37

*Means followed by the same letter in a column do not differ by Scott-Knott test at 5% probability*.

* *coefficient of variation*.

Effect (*P* < 0.05) of hybrids was observed for the DMY of the first cut, which ranged from 6,592.17 to 14,540.23 kg/ha. Two groups were formed: the more productive group, with yields of 9,471.32 to 14,540.23 kg/ha, and the less productive group, with yields of 6,592.17 to 9,077.50 kg/ha. In the second cut, however, the DMY showed no differences (*P* > 0.05) among the hybrids, varying between 2,600.53 and 5,942.66 kg/ha, with an average of 3,339.84 kg/ha.

Table 6 shows the average AFMY and ADMY from the two cuts for the hybrids in question. For the AFMY, the values differed (*P* < 0.05) among the hybrids, forming two groups, with an average of 46,997.21 kg/ha. The group with the highest productivity had mean values ranging from 57,263.47 to 47,145.32 kg/ha, whereas the group with the lowest productivity had an average yield varying from 44,620.57 to 34,512.01 kg/ha. The average ADMY also differed (*P* < 0.05) among hybrids, forming two groups. The higher group had mean values ranging from 18,003.00 to 14,221.94 kg/ha. For the lower group, the mean values ranged from 13,318.41 to 9,902.43 kg/ha.

## Discussion

The mean value of all hybrids for the number of tillers (NT) in the first cut (163,640.75) was similar to those found by Botelho et al. (2010), with the exception of hybrid 17, which reached 203,801.67 plants per hectare. In both studies, there was an increase in the number of tillers in the second cut, which can be explained by the lower rate of tiller mortality and increased tillering; tillering was likely stimulated after the first cut, suggesting that all of the plants sprouted, with the emergence of one or more tillers (Table 3). In the current study we obtained a greater number of tillers (NT) than Molina et al. (2000), who evaluated 6 sorghum genotypes and found that genotypes AG2006, CMSXS756, BR601, BRS701, BR303, and BR304 produced 106,00; 98,000; 87,000; 96,000; 105,000; and 99,000 plants per hectare, respectively. Following the recommendations of EMBRAPA Milho e Sorgo, at thinning, considering the row spacing, a stand of 170,000 plants/ha was left. Thus, the difference between the stand at harvest and the stand at thinning can be interpreted as a loss, representing plant mortality, a trait of great importance in the selection and characterization of hybrids.

**Table 3:** Mean values for the number of tillers per hectare (NT), plant height (PH), and stem diameter (SD) in the first and second cuts of 24 sorghum hybrids cultured in a semiarid region.

**Hybrids**	**NT**		**PH**		***SD***	
	**Cut 1**	**Cut 2**	**Cut 1**	**Cut 2**	**Cut 1**	**Cut 2**
944007	122,281.00	163,041.33	1.86b	1.76a	1.84b	1.09
944056	167,836.67	189,415.67	1.89a	1.98a	1.67b	1.39
944040	158,246.00	194,211.00	1.95a	1.91a	2.21a	1.24
944009	165,439.00	184,620.33	2.02a	1.98a	2.10a	1.19
945015	175,029.67	203,801.67	1.98a	1.82a	1.93b	1.10
945019	172,632.00	155,848.33	1.79b	1.60b	1.65b	1.35
945020	155,848.33	187,018.00	1.90a	1.64b	2.15a	1.23
945023	127,076.33	170,234.33	1.82b	1.35b	1.87b	1.23
945026	155,848.33	194,211.00	1.54b	1.35b	2.10a	1.23
945021	143,860.00	129,474.00	1.77b	1.63b	2.24a	1.34
945027	167,836.67	210,994.67	1.57b	1.59b	1.78b	1.28
945022	172,632.00	230,176.00	1.60b	1.54b	1.88b	1.10
944043	158,246.00	191,813.33	2.15a	1.86a	2.05a	1.33
944033	189,415.67	194,211.00	1.66b	1.87a	1.90b	1.28
944034	158,246.00	175,029.67	2.01a	1.88a	1.75b	1.26
946007	189,415.67	172,632.00	1.86b	1.81a	1.88b	1.13
946015	203,801.67	196,608.67	2.20a	1.94a	1.88b	1.16
946016	165,439.00	206,199.33	2.12a	1.78a	2.10a	1.16
946013	148,655.33	206,199.33	1.95a	1.90a	1.94b	1.24
946042	177,427.33	191,813.33	2.03a	1.91a	1.64b	1.35
946043	175,029.67	170,234.33	2.12a	1.85a	1.65b	1.24
BRS 655	172,632.00	191,813.33	1.73b	1.73a	1.67b	1.29
Volumax	110,292.67	134,269.33	2.06a	1.59b	2.35a	1.35
BRS 610	194,211.00	160,643.67	1.82b	1.76a	1.95b	1.30
Mean	163,640.75	183,521.40	1.89	1.75	1.92	1.24
CV^*^ (%)	22.96	20.84	14.91	9.59	13.12	11.82

*Means followed by the same letter in a column do not differ by Scott-Knott test at 5% probability*.

* *coefficient of variation*.

Silva et al. (2011) evaluated 25 sorghum hybrids in the Paraíban Agreste and verified an average PH of 2.07 m, which was slightly above that observed in the present study. Genotypes Volumax and BRS 610 used in the present study showed lower mean values compared with those from the study conducted by Botelho et al. (2010) under both the first- and second-cut conditions. Monteiro et al. (2004) states that although PH is an important characteristic for forage-sorghum biomass yield, a greater height does not always imply higher DMY, and this relationship is especially dependent on the purposes for which the genotypes have been selected because genotypes with an increased percentage of panicles tend to be shorter. However, for most of the hybrids examined in the present study, the stems were at a higher proportion than panicles (Table 4).

**Table 4:** Mean values of the leaves, stems and panicles of 24 sorghum hybrids based on dry matter in the first and second cuts in a semiarid region.

**Hybrids**	**Leaves**		**Stems**		**Panicles**	
	**In g/kg for DM**					
	**Cut 1**	**Cut 2**	**Cut 1**	**Cut 2**	**Cut 1**	**Cut 2**
944007	151.8b	141.6b	455.7b	641.7	392.5a	216.8
944056	94.9c	120.6b	617.5a	620.2	287.6b	259.2
944040	146.6b	141.0b	483.4b	628.2	370.0a	230.7
944009	124.1c	134.7b	606.1a	636.6	269.8b	228.6
945015	103.5c	140.9b	622.4a	587.5	274.1b	271.6
945019	96.2c	147.8b	552.2a	522.0	351.5a	330.3
945020	82.2c	165.5b	501.6b	586.7	416.2a	247.8
945023	115.2c	195.1a	547.6a	574.5	337.2a	230.4
945026	165.8b	139.0b	458.4b	567.0	375.7a	294.0
945021	322.8a	160.6b	322.8b	612.1	354.3a	227.3
945027	194.0b	136.1b	488.5b	548.4	317.5b	315.6
945022	197.7b	146.0b	414.9b	546.3	387.4a	307.6
944043	147.2b	139.8b	451.0b	595.9	401.8a	264.4
944033	135.0b	137.3b	524.1b	619.4	340.9a	243.2
944034	94.1c	120.8b	618.8a	559.4	287.1b	319.8
946007	124.0c	133.9b	534.1a	602.6	341.9a	263.5
946015	155.4b	139.5b	504.4b	692.8	340.2a	167.7
946016	102.7c	131.7b	633.6a	612.5	263.7b	255.8
946013	115.8c	125.2b	628.4a	613.5	255.7b	261.3
946042	147.5b	131.6b	561.3a	634.6	291.1b	233.8
946043	166.8b	148.8b	572.6a	634.3	260.6b	216.9
BRS 655	100.1c	138.6b	617.7a	622.7	282.3b	238.7
Volumax	112.6c	225.2a	456.5b	602.5	430.9a	172.3
BRS 610	115.6c	144.7b	694.5a	673.8	190.0b	181.5
Mean	138.0	145.3	536.2	605.6	325.8	249.1
CV^*^ (%)	23.97	14.82	16.91	9.52	29.22	26.06

*Means followed by the same letter in column do not differ by Scott-Knott test at 5% probability*.

* *coefficient of variation*.

Agronomic traits such as PH, SD, and NT are variables that can directly affect phytomass yield. The number of tillers is related to the tiller population as a function of the area, whereas SD and HP are related to the volume and/or weight of the tiller. The associations among these agronomic traits can have important effects, such as influencing stand density, the physical size of the tiller, and biomass production per area, which has been confirmed by Goes et al. (2011), who observed that the highest PH and SD values provided greater DMY in sorghum.

Flaresso et al. (2000) discussed nutritional value and considered the stems to be responsible for the low nutritional quality of the silage due to the greater presence of fibers in this material. On the other hand, Zanine et al. (2007) emphasized the importance of the stem in the lactic acid fermentation of the silage because this portion of the plant contains the most part of soluble carbohydrates, which are the main substrate for the lactic acid bacteria, which is responsible for the proper preservation of the silage (Table 5).

**Table 5:** Mean values of the number of leaves (NL), the average leaf size (ALS) and the panicle size (PS) in the first and second cuts of 24 sorghum hybrids cultured in a semiarid region.

**Hybrids**	**NL**		**ALS (cm)**		**PS (cm)**	
	**Cut 1**	**Cut 2**	**Cut 1**	**Cut 2**	**Cut 1**	**Cut 2**
944007	6.17d	6.50b	59.17	42.67	25.50b	21.33
944056	6.83c	7.33a	51.33	46.50	23.00 b	22.83
944040	8.67b	8.17a	62.50	44.33	26.67b	24.67
944009	7.17c	7.67a	57.67	41.00	24.17b	22.33
945015	5.50d	6.67b	68.33	50.00	29.83a	27.83
945019	5.33d	7.67a	56.67	41.00	27.67a	23.50
945020	5.67d	7.83a	65.67	44.67	33.17a	24.17
945023	6.17d	7.67a	57.33	42.83	27.00b	21.17
945026	6.67c	7.00b	58.00	43.83	24.00b	20.17
945021	6.50c	7.83a	59.00	46.33	30.00a	25.50
945027	6.67c	6.00b	60.50	48.50	26.50b	26.00
945022	7.17c	6.67b	63.17	45.50	25.00b	21.67
944043	8.17b	8.00a	65.17	46.33	29.50a	24.33
944033	7.33c	6.67b	54.33	48.33	22.33b	25.50
944034	7.67b	7.00b	57.50	46.83	25.00b	24.17
946007	7.33c	7.00b	60.50	41.50	25.00b	25.00
946015	8.00b	7.50a	63.33	44.67	30.17a	25.00
946016	7.00c	8.00a	54.83	44.50	29.50a	22.83
946013	8.50b	7.33a	45.17	45.17	24.00b	24.83
946042	8.50b	7.50a	51.67	46.33	26.33b	26.17
946043	8.50b	8.50a	58.33	44.67	26.17b	24.67
BRS 655	6.17d	8.67a	52.00	45.67	22.33b	22.33
Volumax	9.83a	7.33a	56.50	56.50	28.33a	23.83
BRS 610	9.33a	7.00b	52.00	50.17	26.33b	27.67
Mean	7.28	7.40	57.94	45.74	26.56	24.06
CV ^*^(%)	11.59	9.50	12.81	8.99	13.08	9.75

*Means followed by the same letter in a column do not differ by Scott-Knott test at 5% probability*.

* *coefficient of variation*.

**Table 6:** Mean values for the accumulated fresh matter yield (AFMY) and accumulated dry matter yield (ADMY) of cuts from 24 sorghum hybrids in a semiarid region.

**Hybrids**	**AFMY kg/ha**	**ADMY kg/ha**
944007	34,512.01b	9,902.43b
944056	43,042.91b	15,189.07a
944040	49,140.18a	12,304.21b
944009	49,073.04a	18,003.00a
945015	51,084.68a	15,345.64a
945019	35,516.63b	10,889.58b
945020	48,521.58a	17,302.56a
945023	43,328.23b	14,703.51a
945026	36,223.94b	10,116.12b
945021	39,259.39b	12,235.19b
945027	43,134.02b	11,736.77b
945022	49,483.04a	12,102.31b
944043	39,614.25b	10,165.23b
944033	57,263.47a	17,001.94a
944034	50,155.58a	16,398.76a
946007	52,748.66a	17,476.48a
946015	50,024.91a	15,569.41a
946016	47,145.32a	14,972.52a
946013	51,135.03a	15,099.31a
946042	52,484.92a	16,852.72a
946043	54,849.02a	15,782.48a
BRS 655	44,620.57b	13,318.41b
Volumax	49,732.40a	14,221.94a
BRS 610	55,839.26a	14,540.27a
Mean	46,997.21	14,217.91
CV (%)	18.33	20.45

Means followed by the same letter in a column do not differ by Scott-Knott test at 5% probability. ^*^ coefficient of variation.

Comparing plant components, Neumann et al. (2002) concluded that the panicle is the sorghum plant component that can determine silage quality because it has the highest concentrations of DM, crude protein (CP) and *in vitro* dry matter digestibility (IVDMD), as well as the lowest levels of fibrous components, compared with the stems and leaves. However, for an effective use of sorghum grain, it is necessary that the panicles be disintegrated or at least broken in the process of forage grinding. According to Silva et al. (2011), greater participation of panicles in sorghum may result in an increased nutritional value for the silage, due to the increased amount of total digestible nutrients.

During the regrowth of sorghum, there was an increased percentage of stems and a decreased percentage of panicle; we can thus assume that there was a possible decrease in the nutritional value of the plant because the panicle is the most nutritious part of the plant, whereas the stem is high in fibers. Additionally, the increased moisture of the sorghum during regrowth can be explained (Table 2) by the stem being the component of highest moisture, while the panicle is responsible for the increase in plant DM for silage. The increased number of stems in the second cut can be explained by the decreases observed in the ALS and PS; thus, the stem component was predominant in the component ratio (Table 4).

According to McDonald et al. (1991), the plant DM content is important in the ensiling process because it is a factor that determines the type of fermentation that will take place within the silo. For the production of good-quality silage, that author suggests that the DM concentration should be greater than 250 g/kg, which was associated with an appropriate level of soluble carbohydrates. França et al. (2011), by evaluating the qualitative characteristics of sorghum hybrid silage, found variations of 216–287 g/kg in the DM content. The average value observed for DM by Colombo et al. (2007) (24.7%), for sorghum genotypes, are closer of recommended for silage. These variations in DM are due to climatic factors and forage cutting point.

The results of the current study were confirmed by Botelho et al. (2010), who demonstrated the potential regrowth capacity of sorghum. The sorghum in the present study produced satisfactory yields in the first and second cuts and was thus suitable for silage production. In general, this characteristic is favorable because it reduces production costs by reducing the costs of labor and seeds for sowing, especially by maximizing the use of the area as other crops become viable for both grain and silage, without the need for replanting (Foloni et al., 2008). This aspect is especially interesting for the semi-arid agricultural system because the rural areas of this region are reduced, thus requiring a high yield per unit area.

Evaluating the DMY of 25 sorghum hybrids in the Paraíba Agreste region (Brazil), Silva et al. (2011) observed DMY values ranging from 7,679.87 to 20,948.70 kg/ha, with an accumulated rainfall slightly above 400 mm. Perazzo et al. (2013) evaluated sorghum genotypes in place similar to the current study and found DMY values ranging from 10.882 to 14.519 kg/ha with rainfall of 115 mm. The values in the second cut for DMY are lower than the 9,000 to 12,000 kg/ha range found by Botelho et al. (2010) during the regrowth period.

It is important to consider the soil-climate conditions observed during the experimental period. Although the region has a semi-arid climate, abnormal levels of rainfall (total 635 mm) occurred during the crop cycle, which caused excessive soil moisture. This high level of water in the soil probably affected the yields, especially of the first cut, during which most of the rain was observed (540–578 mm). Bonfim-Silva et al. (2011) evaluated the initial development of grasses subjected to water stress and showed that sorghum, unlike millet and corn, is tolerant to both flooding and drought conditions. Nevertheless, there was a small drop in shoot dry mass under stress. The high capacity for regrowth observed in sorghum is directly dependent on rainfall. Thus, in microregions with lower precipitation levels, the regrowth yield may be compromised or even become unfeasible due to the end of the rainy season.

According to Rezende et al. (2011), the literature is quite controversial with respect to the data on FMY, given the vast amount of material available in the market and the types of sorghum used (forage, dual purpose or cutting, and grazing), presenting values below and above those obtained in the present study. This type of response varies according to a number of factors such as the fertilization system adopted, the soil type, and the distribution and volume of rainfall. However, besides yield variables, other variables such as plant stand, height, and some morphological characteristics are of great importance, especially in the context of forage conservation for ruminants.

As shown in Table 2, there is a noticeable variation in forage yields among the hybrids and between the cuts; some hybrids have a higher yield in only a single cut. To take advantage of the regrowth of sorghum, a joint evaluation of the cumulative yield of the two cuts is necessary to identify specific genotypes that best exploit the regrowth ability in the forage production system. Botelho et al. (2010), by evaluating the AFMY of the first cut and regrowth, observed accumulations of 88,590.00 and 90,080.00 kg/ha for the genotypes Volumax and BRS 610, respectively. In the present study, lower accumulated values of 49,732.40 and 55,839.26, respectively, were observed for these genotypes. Penna et al. (2010) evaluated different cuts of hybrids developed by Embrapa Milho e Sorgo and observed average accumulated values for the two cuts of 21,850.00 kg/ha for the first season and 36,570.00 kg/ha for the second season, which are lower values than those observed in the current study.

It is worth mentioning that in the present study, the accumulation of forage yields was obtained with a total cycle of 111 days. For semiarid regions, where the rainy season is short (3–4 months), this crop cycle length, including the use of the regrowth, is feasible due to its rapid growth and high productivity, which will therefore reduce production costs and maximize the production system of small farms, as observed by Rao et al. (1999) and Ahmeda et al. (2016). Given the various characteristics presented by the sorghum hybrids, attention should be paid to the forage yields and agronomic characteristics of the plants according to their intended use. These aspects influence the type of varieties for use in direct grazing, fresh cut, regrowth, silage production and haymaking.

## Conclusion

Overall, the hybrids evaluated in the present study combine high yields with high percentages of panicles in the DM.

The sorghum hybrids showed changes in their agronomic characteristics in the different cuts, with an increased percentage of stems and a decreased percentage of panicles in the regrowth period, resulting in a decrease in the nutritional value of the plants.

The sorghum hybrids showed satisfactory accumulations of forage yields due to their high yield after regrowth; therefore, these hybrids are suitable for use in animal production systems in semi-arid regions.

## Author contributions

AP: part of the master's dissertation and responsible for the preparation of the manuscript. GC and ES: teachers responsible for project orientation and statistical analysis. RR, TS, HB, and GP: participated in the data collection of field research. JR: researcher of the Brazilian Corporation of Agricultural Research that provided the seeds and the experimental test.

### Conflict of interest statement

The authors declare that the research was conducted in the absence of any commercial or financial relationships that could be construed as a potential conflict of interest. The reviewer PC and handling Editor declared their shared affiliation, and the handling Editor states that the process met the standards of a fair and objective review.
